# Identification and Positioning of Abnormal Maritime Targets Based on AIS and Remote-Sensing Image Fusion

**DOI:** 10.3390/s24082443

**Published:** 2024-04-11

**Authors:** Xueyang Wang, Xin Song, Yong Zhao

**Affiliations:** College of Aerospace Science and Engineering, National University of Defense Technology, Changsha 410073, China; wangxueyang@nudt.edu.cn (X.W.); zhaoyong@nudt.edu.cn (Y.Z.)

**Keywords:** Automatic Identification System (AIS), optical remote-sensing image, maritime targets, multi-source data fusion and application

## Abstract

The identification of maritime targets plays a critical role in ensuring maritime safety and safeguarding against potential threats. While satellite remote-sensing imagery serves as the primary data source for monitoring maritime targets, it only provides positional and morphological characteristics without detailed identity information, presenting limitations as a sole data source. To address this issue, this paper proposes a method for enhancing maritime target identification and positioning accuracy through the fusion of Automatic Identification System (AIS) data and satellite remote-sensing imagery. The AIS utilizes radio communication to acquire multidimensional feature information describing targets, serving as an auxiliary data source to complement the limitations of image data and achieve maritime target identification. Additionally, the positional information provided by the AIS can serve as maritime control points to correct positioning errors and enhance accuracy. By utilizing data from the Jilin-1 Spectral-01 satellite imagery with a resolution of 5 m and AIS data, the feasibility of the proposed method is validated through experiments. Following preprocessing, maritime target fusion is achieved using a point-set matching algorithm based on positional features and a fuzzy comprehensive decision method incorporating attribute features. Subsequently, the successful fusion of target points is utilized for positioning error correction. Experimental results demonstrate a significant improvement in maritime target positioning accuracy compared to raw data, with over a 70% reduction in root mean square error and positioning errors controlled within 4 pixels, providing relatively accurate target positions that essentially meet practical requirements.

## 1. Introduction

In the contemporary maritime environment, the complexity of maritime traffic underscores the significance of researching the identification of abnormal ship targets at sea. Maritime abnormal targets may include unknown targets not broadcasted by satellite positioning systems (e.g., AIS). Identifying maritime targets is of vital importance for ensuring maritime traffic safety, responding to emergencies, preventing illegal activities, and achieving effective resource management [1,2]. There are many limitations to using a single data source in the identification of abnormal ship targets at sea, primarily in the limited coverage and dimensionality of the target information, which restricts the in-depth understanding of the target. Satellite remote-sensing imagery, as a commonly used data source, can provide the location and shape characteristics of the target, but its information is still limited, and it is difficult to obtain detailed identity information about the target. To make up for the shortcomings of a single data source, multi-source data fusion has become an effective solution.

The Automatic Identification System (AIS) is widely deployed on maritime vessels, achieving extensive coverage in maritime areas. It plays a significant role in maintaining maritime safety, as well as in planning and managing maritime traffic, among other scientific research aspects [3,4,5]. AIS data is a commonly used auxiliary data source when studying maritime targets. In combination with radar data [6,7,8], SAR data [9,10,11], and optical remote-sensing image data [12,13], fusion has been widely employed and plays a crucial role in various fields. The AIS transmits the dynamic information of the ship, including but not limited to the ship name, call sign, speed, heading, destination, and so on through radio communication. This information serves as a reliable foundation for identifying abnormal ship targets at sea [14]. The fusion of satellite images and AIS data can improve the accuracy and comprehensiveness of target identification by considering static appearance and dynamic behavior.

Moreover, the AIS also uses GPS to obtain the ship position information. Each AIS-equipped ship is equipped with a GPS receiver to acquire satellite signals and determine the exact position of the ship [15]. AIS data that can provide accurate geolocation can also be used as control points in oceanic areas to improve the accuracy of optical satellite positioning of ship targets under uncontrolled conditions. Obtaining the precise position of ship targets has important strategic, security, and economic significance in the naval and nautical fields. Traditional correction methods for positioning errors in satellite remote-sensing images usually rely on ground control point data acquired from calibration fields [16]. However, for maritime areas, it is very difficult to obtain valid ground control points. There are some correction methods that utilize public geographic information and do not require ground control points data. By using auxiliary data such as publicly available digital elevation models, or by using multiple images to match points of the same name, the position, size, and other information of known target points are obtained, so as to realize the correction of target positioning errors [17,18]. However, the application of these uncontrolled methods is somewhat limited due to complex challenges, such as dynamic changes in the sea surface and uncertainty in the quality of the geographic data. In this context, multi-source data fusion has likewise become an effective method to improve the accuracy of target positioning at sea.

In summary, the fusion of AIS data and optical remote-sensing images allows for complementary advantages between the datasets, thereby enhancing the efficiency and accuracy of maritime target monitoring. It serves as a reference for research on more sensors and higher-level data fusion technologies, further advancing maritime situational awareness. Additionally, the fusion of maritime targets based on the AIS data and optical remote-sensing images is of significant importance for achieving satellite-based abnormal target identification and positioning correction. By analyzing the fusion results of the two datasets, the identity information of targets can be obtained, enabling the identification of abnormal targets in maritime areas. Moreover, the positional information provided by the AIS can serve as maritime control points, improving target-positioning accuracy and reducing reliance on ground-based error correction, facilitating rapid on-orbit applications. With a development strategy shifting focus from coastal regions to the open ocean, ship-target identification and positioning technology based on the fusion of the AIS and optical images will play a crucial role in oceanic observation.

## 2. Materials and Methods

This paper explores the application of maritime target fusion in the fields of identity recognition and positioning based on two data sources: the AIS and remote-sensing images (Changguang Satellite Technology Co., Ltd., Changchun, China). The innovation is mainly manifested in two aspects. Firstly, the fusion method comprehensively considers the multidimensional feature information of targets from both datasets, enhancing the distinguishability between targets, and thereby, resulting in more accurate fusion results. Secondly, addressing the challenge of geometric correction in remote-sensing images of the open sea, this paper proposes an innovative approach. By introducing the AIS data (China Communications and Information Center-myships.com, Beijing, China) as control points, it successfully solves the problem of geometric correction in marine surveying remote-sensing images, thus improving the accuracy of maritime target positioning.

The general implementation process of the proposed method is as follows: Initially, preprocessing is applied to both datasets to extract multi-dimensional features of ship targets. The common features shared by both datasets, including position, heading, and size, are selected as fusion features to align the features and coordinate systems. Subsequently, using the Coherent Point Drift algorithm, the preliminary association between targets from the two datasets is achieved based on position features. After introducing attribute features, target fusion from both datasets is accomplished through fuzzy comprehensive decision-making to identify the same target from disparate sources, obtaining a comprehensive description of the target. Finally, using AIS-provided target positions as control points, they are incorporated into the established positioning error correction model to enhance the positional accuracy of maritime targets in remote-sensing images, simultaneously obtaining precise positioning along with ship-identity information.

### 2.1. Data Preprocessing

#### 2.1.1. Optical Remote-Sensing Image Object Detection Based on RTMDet

**Figure 1:**
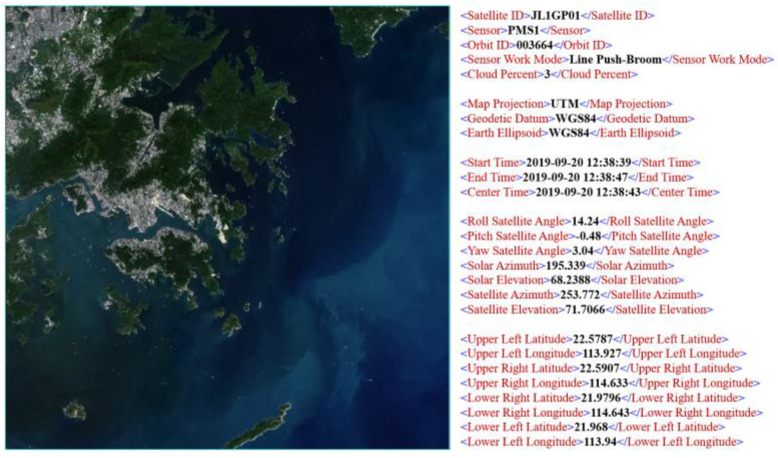
Single image from the Jilin-1 satellite and its related information.

The optical remote-sensing images used in this study are sourced from the Jilin-1 satellite, specifically the Jilin-1 Spectral-01 satellite. This satellite was successfully launched on 21 January 2019, from the Jiuquan Satellite Launch Center. The technical specifications of the satellite are detailed in Table 1. The Spectral-01 satellite is equipped with various payloads, including a multispectral imager, short-wave, mid-wave, and long-wave infrared cameras, along with an onboard intelligent processing system. This configuration allows the satellite to capture remote-sensing data with a resolution of 5 m and to cover 26 different spectral bands. The experiment utilized a remote-sensing image of the waters surrounding Hong Kong, taken by the Jilin-1 satellite on 20 September 2019. The spatiotemporal information and related auxiliary data associated with this image are illustrated in Figure 1.

**Figure 2:**
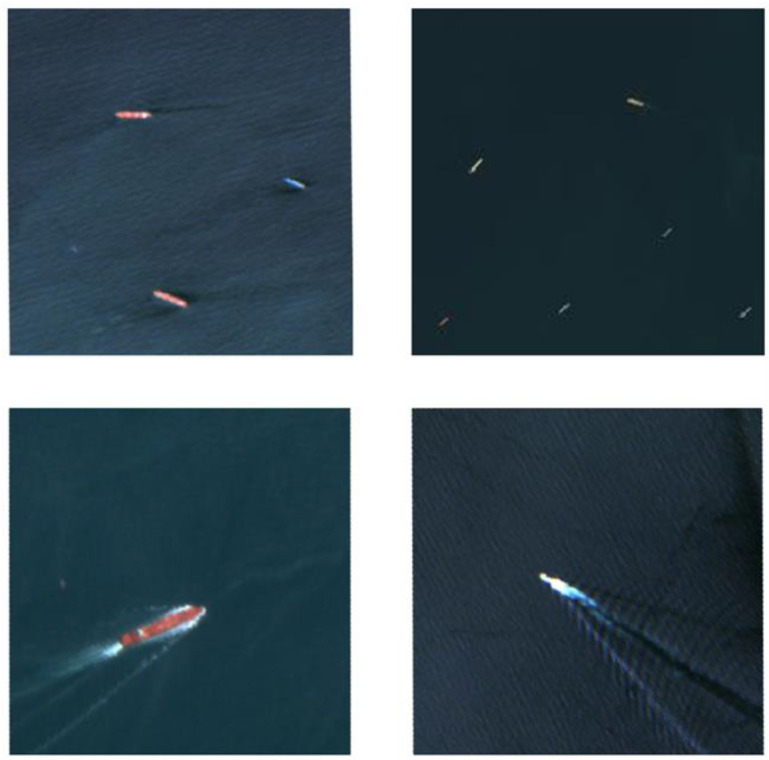
Examples of images in the dataset.

Due to the significant size differences among various types of vessels in the remote-sensing image, including the possibility of smaller-sized ships, and considering the image resolution of the used remote-sensing image, this paper chooses to train the network by autonomously constructing a dataset. Data augmentation is a commonly used technique for self-building datasets, not only helping alleviate overfitting issues but also enhancing the network’s robustness to various scenes and transformations [19,20]. By applying diverse transformations such as rotation, scaling, zooming, gaussian blur, etc., the dataset becomes more enriched and diversified. To ensure the effectiveness of training, the number of images in the dataset is expanded to over 2000, and examples of images from the dataset are shown in Figure 2.

**Figure 3:**
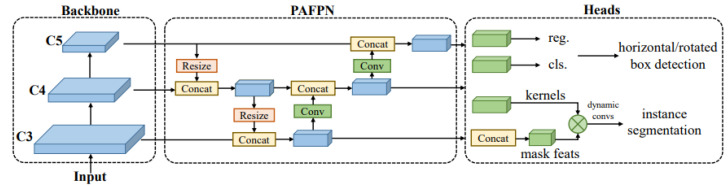
Component structure of the RTMDet network.

In maritime scenarios, ships may appear in various postures and rotational angles, such as navigating, anchoring, or tilting due to wind and waves. Rotation target detection can more effectively capture crucial information about the orientation of maritime ship targets, providing a more comprehensive source of information for subsequent target fusion and result analysis. When addressing the task of rotation target detection, the choice of algorithm is crucial, and RTMDet stands out among many algorithms with its unique design and efficient performance [21]. One of its core advantages lies in the clever use of large-kernel depth convolution. By introducing this design into the backbone and neck, the model can comprehensively capture the features of rotating targets, improving the accuracy of detection. The structure of the RTMDet network is illustrated in Figure 3. The rotating bounding box plays a key role in the research process, as a pre-step to obtain important feature information such as target size and orientation, which provides an accurate positioning and identification basis for the whole target detection process. The RTMDet network will finally output the information content such as the position, type, and confidence of the target.

#### 2.1.2. AIS Data Processing

Usually, the AIS includes static information, dynamic information, and voyage information, such as the ship name, ship type, latitude, longitude, speed, and heading information, etc., and the specific information covered can be seen in Table 2. Due to factors such as equipment malfunctions, signal interference, configuration issues, forgery, or improper operations, received AIS data may present various errors, including data missing, duplication, and anomalies. To ensure the accuracy and reliability of the AIS data in practical applications, the preprocessing stage involves filtering out these erroneous values.

The receiving range of the satellite-based AIS is generally over a thousand kilometers, far exceeding the coverage of remote-sensing images. This enables the satellite-based AIS to simultaneously cover large maritime areas, monitor the real-time positions of numerous vessels, and acquire relevant information. In contrast, remote-sensing images are constrained by the field of view of the satellite camera and the satellite’s orbit, allowing them to capture images of only specific regions at a time. To ensure the temporal and spatial consistency between the AIS data and remote-sensing images, it is necessary to perform temporal and spatial calibration before the formal fusion [22]. In spatial calibration, the AIS data needs to be filtered based on the imaging geographic range of the remote-sensing image. This involves aligning the image coordinates with the coordinates provided by the AIS for target points and correcting the installation position of the GPS antenna with the centroid position extracted in the preprocessing stage. For temporal calibration, the steps include selecting the AIS data within a certain time range before and after the imaging-center moment. Based on the existing trajectory sequence of ships, the position and heading values of a vessel at the imaging-center moment are predicted. Temporal and spatial calibration is a crucial preprocessing step for heterogeneous data, eliminating errors caused by differences in data sources and characteristics, thereby enhancing the efficiency of subsequent data fusion and analysis.

#### 2.1.3. Feature Extraction and Alignment

**Figure 4:**
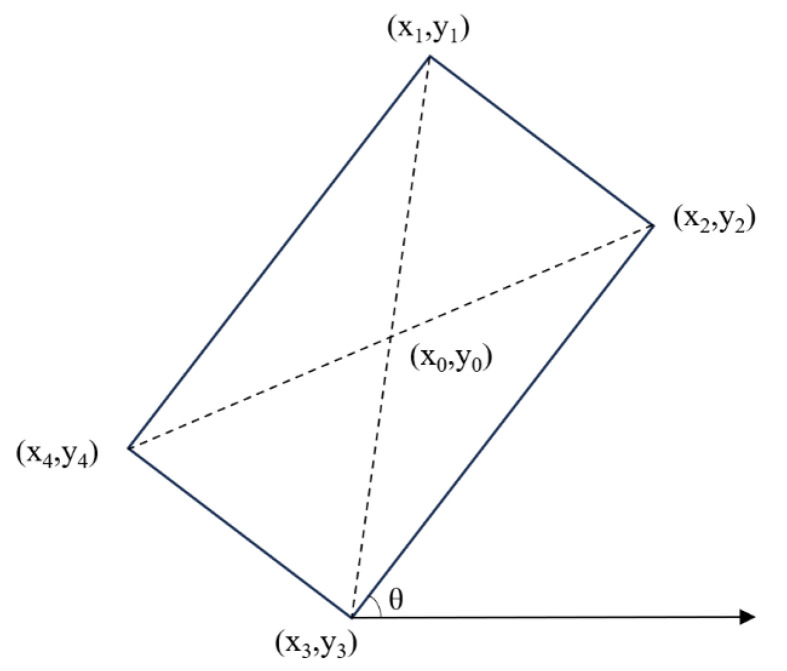
Schematic of the output rotating bounding box.

The essence of data fusion is to establish connections between two heterogeneous data sources at the same moment, same geographical position, and same target. This implies that after extracting feature information from remote-sensing images, there is a need to align the common fusion features from both sources, ensuring their consistency in describing the features of ship targets. The AIS data contains a diverse set of target information. In comparison, features extracted from remote-sensing images are relatively limited and mainly rely on ship size, heading, and geographical position information obtained through rotating object detection boxes. The ship-target features common to both data are position, size, and heading. The image data is passed through the target-detection network, and the schematic of the output rotated bounding box can be seen in Figure 4 below.

(1) Target heading feature extraction and alignment

The angle of the rotating bounding box provides information about the heading of the target. By measuring the angle of the rotating bounding box relative to the horizontal direction, the orientation of the ship relative to the image can be determined. Heading is one of the important features of a ship target in a remote-sensing image, and it is crucial for the analysis of the heading and the judgment of its behavior. In Figure 4, the acute angle of the long side of the rotating bounding box relative to the positive direction of the *x*-axis is *θ*. The formula for the direction *θ* of the rotating box is as follows:

(1) $$\theta =\arctan(\frac{y_{1}-y_{4}}{x_{1}-x_{4}})$$

The angle *θ*₀ represents the angle of the remote-sensing image itself relative to the true north direction in the WGS84 coordinate system. The final calculation formula for the heading *θ*₁ is as follows:

(2) $$\theta_{1}=\theta_{0}+\theta$$

(2) Target size feature extraction and alignment

The width and height of the rotating bounding box provide information about the dimensions of the target. The coordinates of the four vertices of the rotating bounding box are (*x*_1_, *y*_1_), (*x*_2_, *y*_2_), (*x*_3_, *y*_3_), and (*x*_4_, *y*_4_), and the length and width of the rotating box can be approximately regarded as the length and breadth of the ship target under the image coordinate system. In order to ensure their consistency with the ship size units provided in the AIS data, the row spatial resolution and column spatial resolution of the remotely sensed image need to be considered. *P* is the spatial resolution of the image, and the actual length and breadth of the final ship target are calculated by the following formula:

(3) $$\begin{array}{l} length=\sqrt{{\left(x_{1}-x_{4}\right)}^{2}+{\left(y_{1}-y_{4}\right)}^{2}}\times P\\ width=\sqrt{{\left(x_{2}-x_{1}\right)}^{2}+{\left(y_{2}-y_{1}\right)}^{2}}\times P \end{array}$$

(3) Target position feature extraction and alignment

The pixel position of the ship-target center point (*x*_0_, *y*_0_) can be calculated by rotating the coordinate positions of the four vertices of the bounding box with the following formula:

(4) $$\begin{array}{l} x_{0}=\frac{x_{1}+x_{2}+x_{3}+x_{4}}{4}\\ y_{0}=\frac{y_{1}+y_{2}+y_{3}+y_{4}}{4} \end{array}$$

### 2.2. Data Fusion

The process of target association and fusion typically involves considerations of both spatial position features and attribute features. In cases where spatial relationships play a significant role in target association, prioritizing the use of position information is advantageous. However, in certain scenarios, relying solely on position information may not be sufficient to distinguish targets with close proximity but different attributes. Introducing attribute features can provide richer and more discriminative target information, aiding in addressing some challenges in target association. Association is a prerequisite for fusion, and fusion algorithms can be used to analyze and evaluate preliminary association results. 

#### 2.2.1. Preliminary Target Association Based on Position Feature

Point-set matching is a method to establish an association or correspondence between two or more datasets by finding points in them that correspond to each other and is widely used in the fields of computer vision, various types of image processing, and pattern recognition. In this framework, each ship target is represented in the point set as a unique point that is at the center moment of image imaging. The key challenge in point-set matching is determining the correspondence between two point sets. To measure these correspondences, the Coherent Point Drift (CPD) algorithm introduces the concept of probability values, where higher probability values indicate higher certainty in the correspondence. The core idea of the CPD algorithm involves introducing a Gaussian Mixture Model (GMM) as a probability density function to model the probability relationships between point sets [23,24,25]. This approach provides flexibility to adapt to the spatial topology of point sets, enabling the algorithm to handle various complex scenarios, including non-rigid deformations, target deformations, and noise. 

Assuming the target point set is *X* = {*x*_1_, *x*_2_, …, *x_N_*}, and the reference point set is *Y* = {*y*_1_, *y*_2_, …, *y_N_*}, where *x_i_* and *y_j_* are the positional coordinates of the corresponding ship targets in sets *X* and *Y*, respectively. Taking the points in the reference set *Y* as the centroids of the GMM, the probability density function can be represented by the following formula:

(5) $$\left\{\begin{array}{l} p(x)=\sum_{m=1}^{M}P(m)p(x|m)\\ p(x|m)=\frac{1}{{\left(2\pi \sigma^{2}\right)}^{D/2}}\exp^{-\frac{\left\|x-{\left.y_{m}\right\|}^{2}\right.}{2\sigma^{2}}} \end{array}\right.$$

where *p*(*x*|*m*) denotes the probability density of the *m*-th Gaussian component.

The objective function of the CPD algorithm consists of two parts: the squared error term for point-set alignment and the regularization term. The inclusion of the regularization term balances the point-set alignment and the model complexity in the optimization process, which helps to control the complexity of the transformation matrix, making it more general and preventing overfitting. The objective function can be expressed as follows:

(6) $$E(R,t)=\frac{1}{2}\sum_{i,j}P_{ij}\left\|R\cdot x_{i}\right.+t-{\left.y_{j}\right\|}^{2}+\frac{\beta }{2}Tr(R^{T}R)$$

where *β* is the regularization parameter, *Tr* (·) denotes the trace operation of the matrix, *R* is the transformation matrix for, *t* is the translation vector, and *P* is the probability matrix.

#### 2.2.2. Target Fusion Incorporating Attribute Features

The attribute features of ship targets are generally relatively stable. Combining them with the existing position features can complement each other, significantly enhancing the overall effectiveness of the strategy. Adopting this integrated approach aims to achieve the final fusion of targets from the AIS data and image data through fusion decisions. This approach indirectly verifies the accuracy of the initial target association results, providing strong support for a comprehensive understanding of the targets. Among the many fusion strategies, the fuzzy integrated decision-making method has attracted much attention for its ability to handle uncertainty and ambiguity. The fuzzy comprehensive decision-making method, by introducing fuzzy logic thinking, considers the fuzziness of fusion results, making it advantageous in addressing complex uncertainty issues in practical applications [26,27,28]. The specific steps for target fusion based on the AIS data and image data are as follows:

(1) Build the factor set

In the common feature vector of the AIS and image data, each type of feature represents an independent factor influencing the fusion algorithm. Therefore, the feature vector {position, heading, ship width, ship length} forms the factor set. The constructed factor set *A* is as follows:

(7) $$A=\{a_{position},a_{heading},a_{width},a_{height}\}$$

(2) Build the weight set

The weight set is used to represent the relative importance of each factor or attribute for the final comprehensive decision. The weights determine the contribution of each factor in the overall assessment, indicating their impact on the overall decision. The specific weights for each factor can be determined through subjective judgment, expert experience, and other methods. The constructed weight set *W* is as follows:

(8) $$W=\{w_{position},w_{direction},w_{width},w_{height}\}$$

(3) Build the evaluation set

The evaluation set is used to assess the degree of target fusion, playing a crucial role in fuzzy comprehensive decision-making. Considering the characteristics of the data and the requirements of the results in this paper, the constructed evaluation set *B* is determined as follows:

(9) B={Fusion success,only image data exists,only AIS data exists}

(4) Determine the fuzzy matrix

The fuzzy matrix is a tool in fuzzy mathematics used to represent fuzzy relations. In fuzzy comprehensive decision-making, the fuzzy matrix is commonly used to indicate the degree of matching between different factors. In the fuzzy matrix, each element’s value is not a traditional precise numerical value but a fuzzy numerical value belonging to the [0, 1] interval. Each row or column in the fuzzy matrix typically represents the fuzzy relationship between one element and other elements, expressing the fuzzy similarity or membership degree between things. The fuzzy matrix *R* composed of the similarity of the *i*-th target in the AIS data and the similarity of the characteristic factors of all targets in the image data is as follows:

(10) $$R={\left[\begin{array}{cccc} r_{position}(pos_{i}^{AIS},pos_{1}^{image}) & r_{direction}(\theta_{i}^{AIS},\theta_{1}^{image}) & r_{height}(w_{i}^{AIS},w_{1}^{image}) & r_{width}(h_{i}^{AIS},h_{1}^{image})\\ r_{position}(pos_{i}^{AIS},pos_{2}^{image}) & r_{direction}(\theta_{i}^{AIS},\theta_{2}^{image}) & r_{height}(w_{i}^{AIS},w_{2}^{image}) & r_{width}(h_{i}^{AIS},h_{2}^{image})\\ \cdots & \cdots & \cdots & \cdots \\ r_{position}(pos_{i}^{AIS},pos_{j}^{image}) & r_{direction}(\theta_{i}^{AIS},\theta_{j}^{image}) & r_{height}(w_{i}^{AIS},w_{j}^{image}) & r_{width}(h_{i}^{AIS},h_{j}^{image}) \end{array}\right]}_{j\times 4}$$

(5) Build the comprehensive evaluation model for integrated judgment

Multiply each feature factor in the rows of the fuzzy matrix *R* by the corresponding feature factor weight values in the weight set *W*, establishing the fuzzy evaluation matrix *B* for the *i*-th target in the AIS data with all targets in the image data:

(11) $$B=R\cdot W={\left[\begin{array}{c} b_{1}\\ b_{2}\\ \cdots \\ b_{j} \end{array}\right]}_{j\times 1}$$

Repeat steps (4) and (5) to establish a fuzzy evaluation matrix for all targets in the AIS data with all targets in the image data. Take the maximum value in matrix *B* as the image data target point successfully fused with the AIS data target point.

### 2.3. Positioning Error Correction

Section 2.2 successfully establishes the link between the target in the AIS and image data and fuses the multi-dimensional feature information of the ship targets. Due to various factors such as sensor attitude errors and atmospheric effects, there are errors in the positioning of targets on remote-sensing images. This section aims to apply the fusion results to the field of positioning, reducing such errors and ensuring high-precision positioning of image data in geographical space. 

The positioning error correction model is the core of the overall process, and its main task is to correct the position of the target in remote sensing by considering multiple sources of error and adopting the form of a mathematical model. In this process, the position information provided by AIS serves as the true position of ship targets, correcting the positioning errors of all targets in the image and updating the coordinates of all targets. The polynomial model is widely used in the correction of positioning errors in remote-sensing images. Its core idea is to model the position error in the image through a polynomial function, whose mathematical expression is [29,30]:

(12) $$P(x,y)=a_{0}+a_{1}x+a_{2}y+a_{3}x^{2}+a_{4}xy+a_{5}y^{2}+\cdots$$

where *P*(*x*, *y*) represents the corrected position, *x* and *y* are the horizontal and vertical coordinates of the pixels in the image, and *a_i_* are the polynomial coefficients obtained through least squares fitting. This model allows for flexible adjustment of polynomial coefficients.

## 3. Experimental Results and Analysis

### 3.1. Data Preprocessing Results

**Figure 5:**
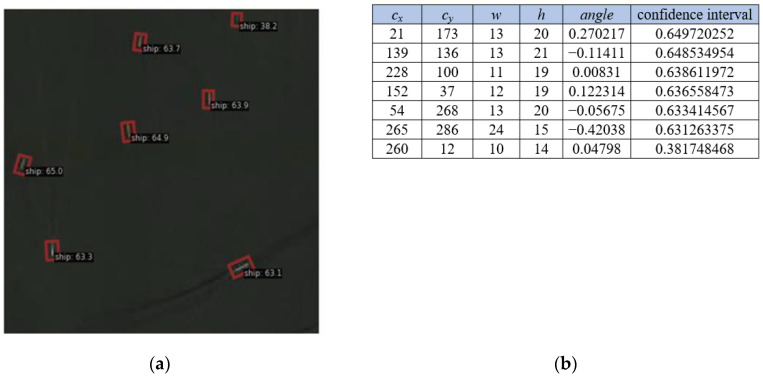
Example of rotating target detection results. (**a**) Image-detection results; (**b**) rotating-bounding-box-detection results.

In deep-learning object detection, network outputs typically include crucial information such as the target’s position, type, and confidence level. Optical remote-sensing images were fed into the RTMDet network, which resulted in 647 ship-target detection results, as shown in Figure 5. This process produced two key output files: one is the picture-detection result and the other is the rotated-target-bounding-box information result. In the picture-detection result, the rotated bounding box is utilized to be able to obtain information about the exact position of the target in the image, the type of target, and the confidence level for this classification. In the rotated-box-information result, the network generates information about the specific position of the target in the image and the confidence level that it belongs to a certain category. The output of the rotated target bounding box was in the form of (c_x_, c_y_, w, h, angle), which consisted of the x-coordinate of the target center point, the y-coordinate of the target center point, the width of the rotated target bounding box, the height of the rotated target bounding box, and the angle of rotation.

**Figure 6:**
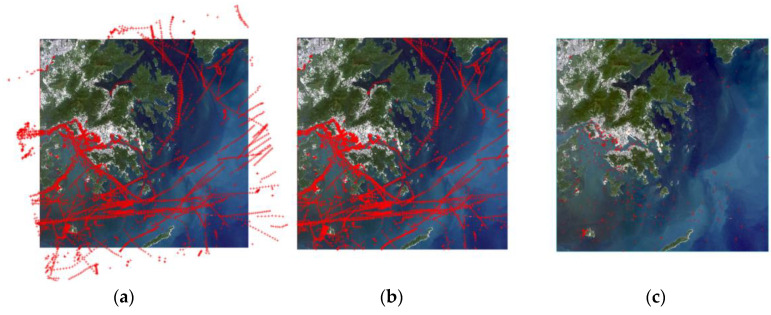
The corresponding results between the AIS data and the remote-sensing image during the preprocessing stage. (**a**) Preprocessing results; (**b**) spatial-calibration results; (**c**) temporal-calibration results.

Currently, only a few satellites are equipped with both optical payloads and AIS devices. Therefore, the AIS data used in this experiment was obtained from Shipxy.com and covers both dynamic and static information, which is synchronized with the remote-sensing images in terms of time and geographic location. Filtering the AIS data within the latitude and longitude range of the four corner points of the remote-sensing image, and consulting the literature combined with the data in this paper, the center moment chosen as the reference is 20 September 2019 12:38:43. Subsequently, further filtering was conducted for the AIS data around the center moment, with a time range of 10 min before and after, specifically from 20 September 2019 12:28:43 to 20 September 2019 12:48:43 AIS data for calibration. Using the remote-sensing image as the base map and visualizing the AIS data as point features, it was observed that the two types of data had achieved a preliminary correspondence. After preprocessing, spatial calibration, and temporal calibration of the AIS data, the effect diagrams of its correspondence with the remote-sensing image are shown in Figure 6 as (a), (b), and (c), respectively, where the red cross dots denote the target positions provided by the AIS, which will be used as the data source for target fusion with the remote-sensing image. After the complete processing workflow, a total of 265 AIS data were retained, at which time each ship target had only one trajectory point at the center moment of remote-sensing image imaging.

### 3.2. Data Fusion Results

#### 3.2.1. Preliminary Association Results Based on Position Feature

Since the number of ship-target position information from the AIS data was less than that from the image data, the ship-target point set from the AIS data was chosen as the reference point set when associating targets using the CPD algorithm. This strategy aimed to let the ship-target point set from the image data with a larger number be the target point set so that it gradually approached the reference point set to realize the effective association between the two targets. After the AIS data preprocessing in Section 2.1.2 and the feature alignment work in Section 2.1.3, the position coordinates of the targets in the two datasets were unified and were in the UTM projected coordinate system, which facilitated the calculation of the distance between the point pairs.

**Figure 7:**
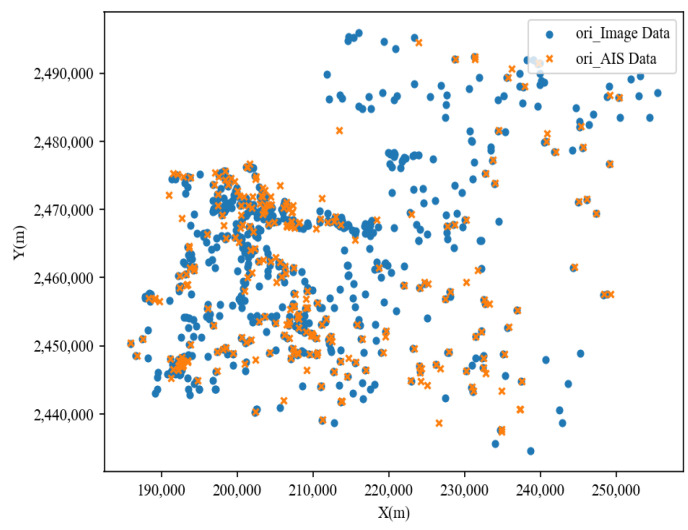
Scatter plot of ship targets in the AIS and image data before position correlation.

The scatter distribution of ship targets in the AIS and image data before position association is shown in Figure 7. Through the scatter distribution plot, it can be observed that the distribution characteristics of ship targets in the AIS and image data were roughly similar. The distribution of the ships in the shore area was relatively centralized, and the distribution of the ships in the area far away from the shore was relatively dispersed. The plot already shows many points exhibiting overlapping phenomena. To determine whether these coincident point pairs from the two data sources represent the same ship target and to validate the effectiveness of associating ship targets based on position features, Section 2.2.1 employed the CPD algorithm to establish associations between the two sets of points.

**Figure 8:**
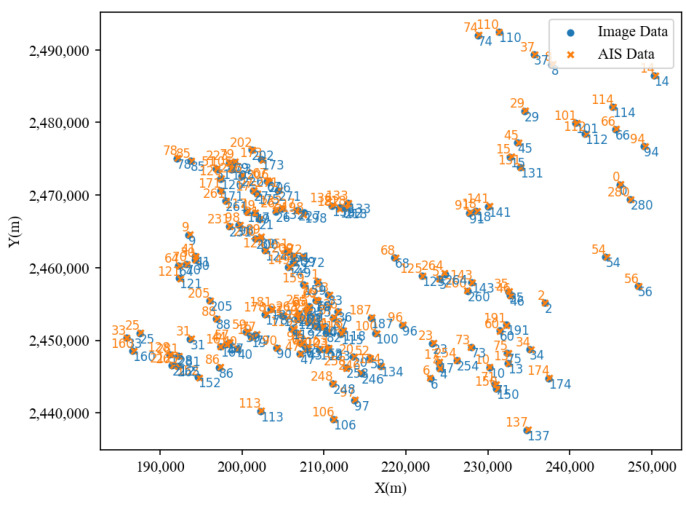
Correlation results obtained using the CPD algorithm.

When associating point sets based on position features, using distance as a threshold condition was an effective choice. If the distance between pairs of points successfully matched by the algorithm was less than this threshold, it was retained; otherwise, it was rejected. Ultimately, the association results obtained using the CPD algorithm amounted to 156 pairs. This indicates that not all points from the AIS dataset have corresponding counterparts in the image data, aligning with the actual scenario. The association results are illustrated in Figure 8, where point pairs with matching numbers indicate that they represented the same ship target.

**Figure 9:**
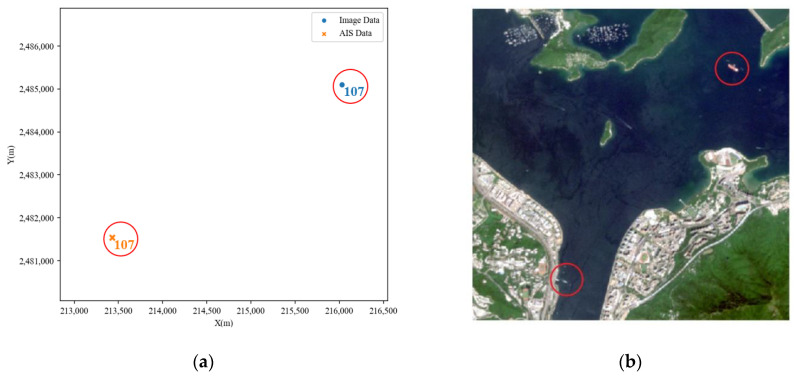
Example of a point pair incorrectly matched despite being distant in position. (**a**) Scatter plot of point pair 107; (**b**) the position of point pair 107 in the actual image.

From observing Figure 8, it can be observed that the successfully matched point pairs by the CPD algorithm are relatively close in distance. However, upon combining with actual remote-sensing images, it was discovered that there are cases of incorrect matching of ship targets. Taking point pair 107 as an example, as shown in Figure 9, it is evident from Figure 9b that these two target points are far apart in the actual image and cannot possibly belong to the same ship target. However, the algorithm results still consider them as successfully matched point pairs. Through the CPD algorithm, each AIS data point is matched to an image data point with the optimal relative position relationship. This does not correspond to the actual situation, as some targets only exist in one of the two types of data and should present a matching failure result. This occurrence suggests that relying solely on positional features for correlation has certain limitations. It is necessary to consider more features of the targets to improve matching accuracy and eliminate incorrectly matched results. At this point, the importance of attribute features becomes particularly prominent.

#### 3.2.2. Target Fusion Results Incorporating Attribute Features

**Figure 10:**
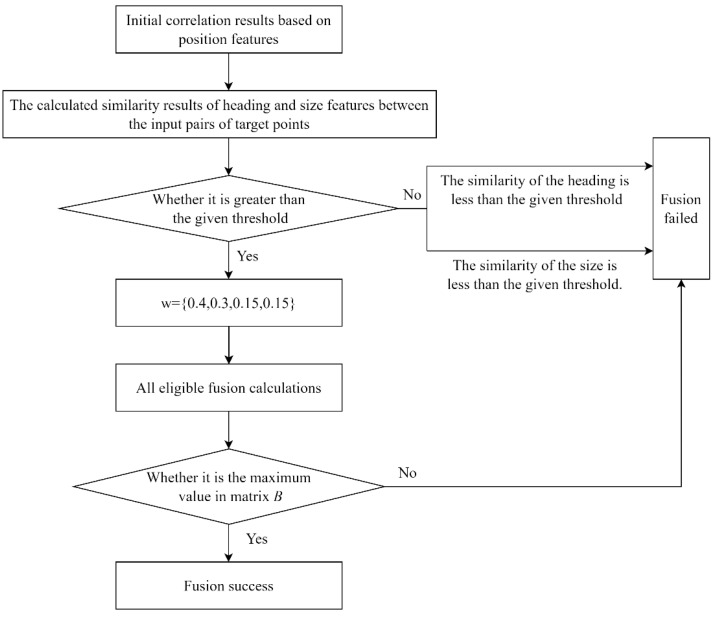
Flow chart of target fusion experiment based on the AIS data and image data.

Introducing the attribute features of ship targets not only could further validate the successful target point pairs matched by the CPD algorithm, but also can add multi-dimensional information to the target points to enhance the differentiation between the targets, improve the matching accuracy, and ultimately realize the target fusion of the AIS data and image data. Using the fuzzy comprehensive decision-making, the target fusion experiment based on the AIS data and image data was successfully implemented, and the overall flow chart is shown in Figure 10.

The specific implementation steps of the target fusion experiment based on the AIS data and image data are as follows:

(1) On the basis of the correlation experiment results based on the ship-target position characteristics in Section 3.2.1, the preliminary correlation results of the target obtained through the CPD algorithm were input.
(2) Determine the priority and value of each feature in the weight set *W*. The priority is set to ensure that the more important features can influence the final fusion result to a greater extent among the information provided by different data sources. The weighting values are ordered as *w_position_* > *w_heading_* > *w_width_* = *w_height_*. Position information is generally considered the most critical and fundamental feature; hence, it is assigned the highest weight value. Direction and size are equally important in some scenarios; heading information is crucial for predicting the target’s motion direction and determining its intentions, while size information aids in distinguishing between different types of targets, particularly between large and small targets.
(3) Although there is a priority among the features, the fusion result must fully consider the influence of all features. Successfully fused target points should be well-matched in all aspects, rather than being considered a successful fusion result solely because of high similarity in a few features, leading to the maximum value in matrix *B*. Therefore, before calculating the fusion result value *b*_i_, it is necessary to scrutinize the similarity situation of each feature between the point pairs and filter out the targets that are more matched in some features but less matched in other features by setting a similarity threshold. Such filtering not only enhances the credibility of fusion results but also aids in identifying more precise reasons for fusion failure.

**Figure 11:**
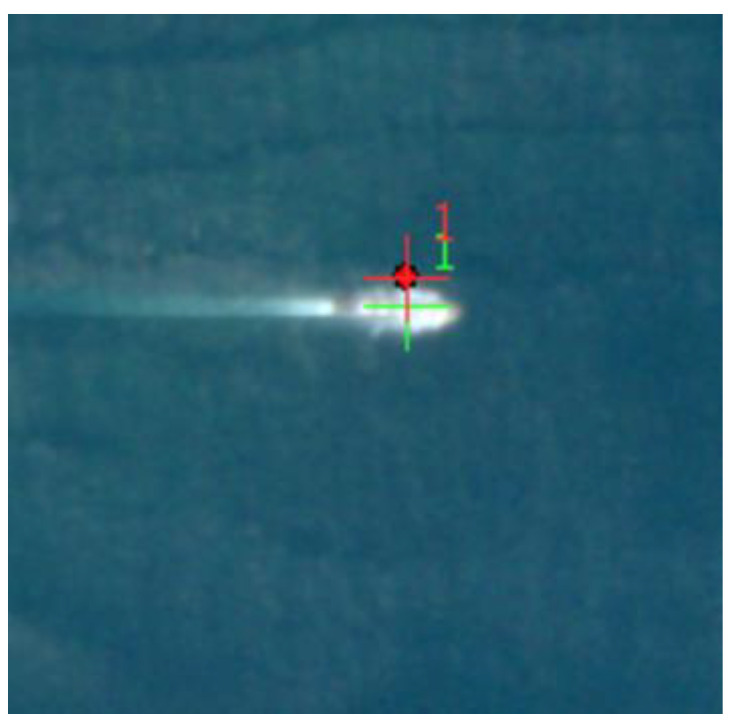
Fusion Success Example.

**Figure 12:**
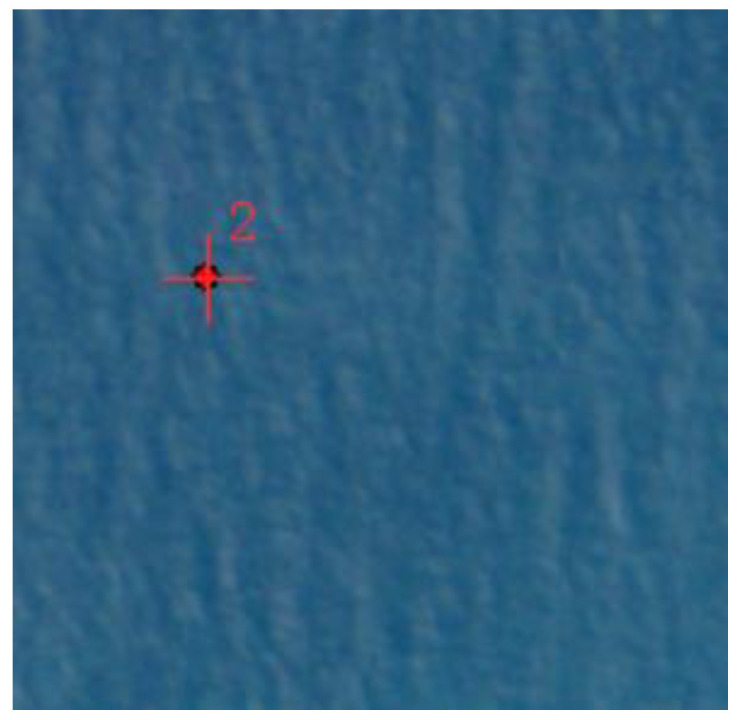
Example of fusion failure with only the AIS data exists.

**Figure 13:**
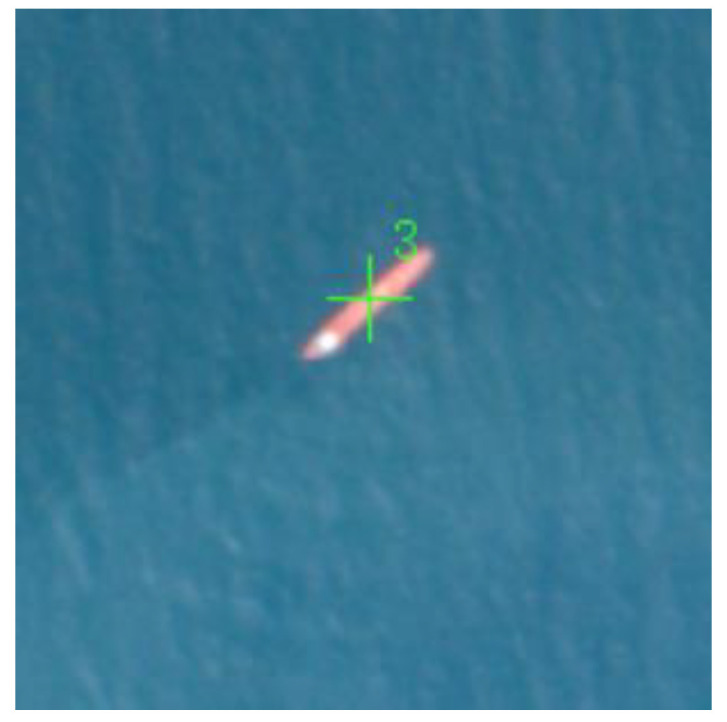
Example of fusion failure with only image data exists.

The success or failure of fusion directly reflects the consistency between the AIS data and image data. Ship-target fusion of the two data sources was accomplished by introducing attribute features, and the final fusion results can be summarized into the following three cases:

(1) The fusion is successful in finding the same ship target from the AIS data and the image data. An example is shown in Figure 11, where the red dots represent the target coordinate points provided by the original AIS, the red crosses represent the target coordinate points provided by the successfully matched AIS, and the green crosses represent the target coordinate points on the successfully matched image. This category means that an effective link between the target in the AIS data and the target in the corresponding remote-sensing image has been successfully established. Through the AIS data, precise target positions, MMSI code, and other identity information were successfully obtained, while image data presented the visual appearance features of the targets, including shape, size, and other visual information. This not only verifies the effectiveness of the fusion method but also provides substantial data support for a comprehensive understanding of the targets. The successful fusion results lay a solid foundation for further analysis and inference, achieving the identity recognition task for the ship target. 

(2) Fusion failed, only the AIS data exists. An example is shown in Figure 12, where the red dots represent the target coordinate points provided by the original AIS and the red crosses represent the target coordinate points provided by the AIS to be matched. The accuracy of the algorithm in detecting ship targets in remote-sensing images is constrained by various factors, including image resolution, background interference, and others. Since not all ship targets can be detected with 100% accuracy in the image, some targets may not pass the detection phase to enter subsequent stages of feature extraction and target association fusion research. If there are a large number of such failures in the fusion results, it may be necessary to consider adjusting the algorithm parameters or trying to replace other target detection networks to improve the detection accuracy of ship targets in remote-sensing images.

(3) Fusion failure, only image data exists. An example is shown in Figure 13, where the green cross represents the target coordinate point on the image to be matched. When encountering such fusion failures, it is crucial to carefully assess whether the AIS equipment aboard the vessel is experiencing anomalies. If the AIS equipment anomaly is due to human shutdown or malfunction, it may indicate potential illegal vessel activity or security risks. In such cases, image data can provide valuable appearance characteristics and positional information of the abnormal target. Utilizing these known features from the image data, relevant authorities can track down vessels that have disabled their AIS equipment and address potential equipment maintenance or communication issues to ensure compliance with safe navigation protocols. Conversely, if the AIS device anomaly involves military vessels, it may be related to military tactics, security protocols, or stealth operations. Understanding the intent behind military vessel activities and recognizing military considerations is essential for distinguishing routine operations from potential security threats. This comprehensive understanding contributes to real-time monitoring, ensuring the security of surrounding waters, and provides vital intelligence for military operations. Ultimately, it offers essential data support for strategic decision-making processes.

In this study, a similarity threshold *r*_0_ is set to 0.6, indicating that the similarity in a specific feature between two targets must reach 60% or higher to be considered potentially the same target and proceed to the next fusion step. If the similarity result in any feature falls below *r*_0_, the initial association result is deemed a fusion failure after secondary verification. The detailed outcomes of the final target fusion results, based on fuzzy comprehensive decision-making, are outlined in Table 3. Out of 156 initial target associations used as input data, 142 passed further verification, successfully consolidating the same ship targets from both data sources. By integrating attribute features, 14 erroneous results were effectively rectified, including 3 cases of heading mismatch and 11 cases of size mismatch. This outcome reaffirms the importance of integrating attribute features into the fusion process. Through the fuzzy comprehensive decision-making process, the accuracy of the final fusion results has been significantly enhanced compared to the results presented in Section 3.2.1. This provides further validation for the necessity of integrating attribute features into the fusion methodology.

**Figure 14:**
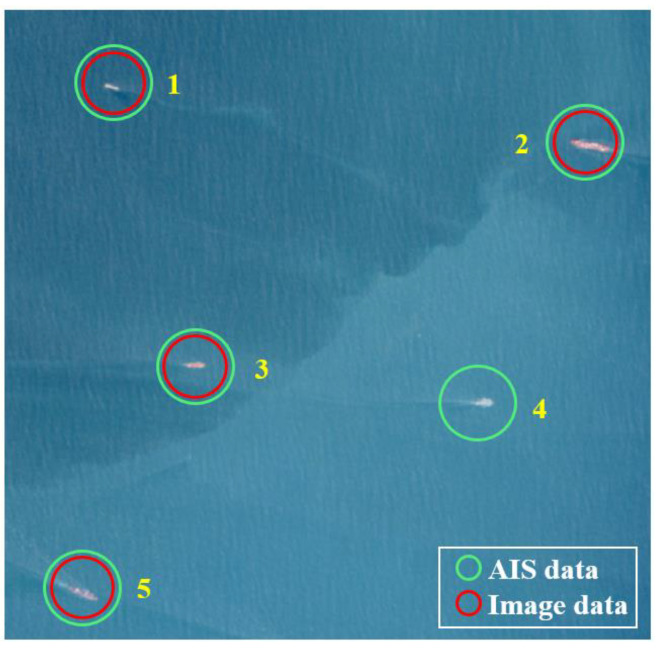
Partial example of ship-target fusion results.

The optical image-based target detection yielded 647 ship-target data while preprocessing of the AIS data retained 283 ship-target data. The significant difference in the quantity of the two types of data resulted in many fusion failures solely based on image data. The main reasons can be summarized as follows: Firstly, the area covered by the optical image is mainly concentrated along the shore, where many ships moored along the shore might not have their AIS turned on, thus failing to receive AIS messages from these vessels. Secondly, during the preprocessing stage of the AIS data, only data within 10 min before and after the imaging-center moment were selected. Due to the frequency of AIS message transmission by these ships, they were not included in the time filtering range and thus were not involved in subsequent processing. Figure 14 shows the fusion results of ship targets in a certain area. Among the five targets, target No. 4 exists solely based on image data and, therefore, fusion fails. Targets No. 1, 2, 3, and 5 have successfully fused, and the various information obtained after fusion can be seen in Table 4.

### 3.3. Positioning Error Correction Results

Among the fused successful point pairs, 50 points evenly distributed throughout the geographic area were selected to participate in the error correction and accuracy analysis work of the target. Based on these 50 successfully fused points, 40 control points are selected to participate in the error correction work, and the remaining 10 are used as checkpoints to verify the accuracy of the results. The accuracy evaluation was performed by calculating the mean error in the *X* and *Y* directions for the checkpoints on the original and corrected remote-sensing image. Subsequently, the root mean square error (RMSE) between the coordinates was calculated to assess the correction accuracy. The formula for calculation is as follows:

(13) $$\left\{\begin{array}{l} RMSE\_x=\sqrt{\frac{\sum_{i=1}^{m}{\left(x_{i}-X_{i}\right)}^{2}}{q}}\\ RMSE\_y=\sqrt{\frac{\sum_{i=1}^{m}{\left(y_{i}-Y_{i}\right)}^{2}}{q}}\\ RMSE=\sqrt{\frac{\sum_{i=1}^{m}\left[{\left(x_{i}-X_{i}\right)}^{2}+{\left(y_{i}-Y_{i}\right)}^{2}\right]}{q}} \end{array}\right.$$

where *q* is the number of checkpoints, *x_i_*, and *y_i_* denote the coordinates of the checkpoints on the original image, and *X_i_* and *Y_i_* denote the coordinates of the checkpoints on the error-corrected image.

**Figure 15:**
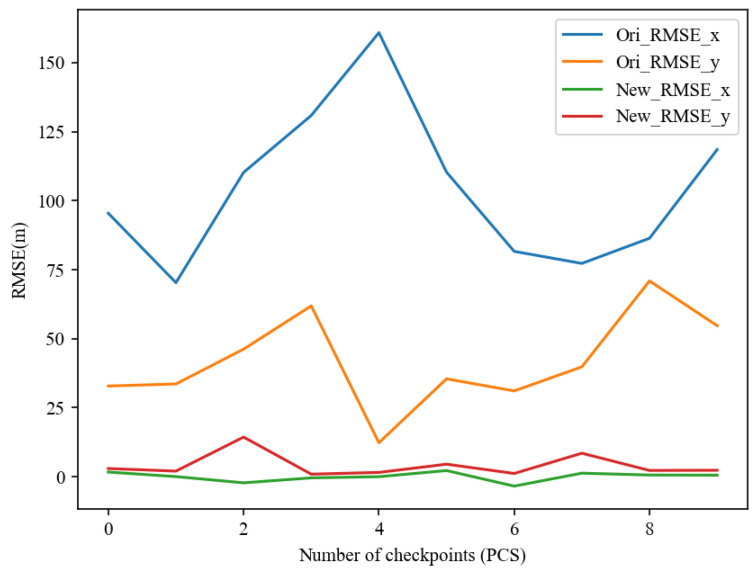
Line chart of deviations in the *x* and *y* directions for 10 checkpoints before and after positioning error correction.

The RMSE after correction using the polynomial model is shown in Table 5. From the table, it can be observed that the maximum RMSE for the checkpoints is 14.52 m, the minimum is 1.01 m, and the average is 4.46 m. The resolution of the remote-sensing image used in this paper is 5 m. Therefore, the root mean square error of the positioning of the 10 checkpoints is within the maximum of 5 pixels, and the average is controlled to be about 1 pixel. The overall root mean square error (RMSE) after error correction is 30.5162 m, which is a 73.5% improvement compared to the pre-correction RMSE of 115.034 m. The deviation line chart for the checkpoints in the *x* and *y* directions before and after correction is shown in Figure 15. This visually demonstrates that the deviation of the 10 checkpoints in the *x* and *y* directions has effectively decreased after the position correction.

**Figure 16:**
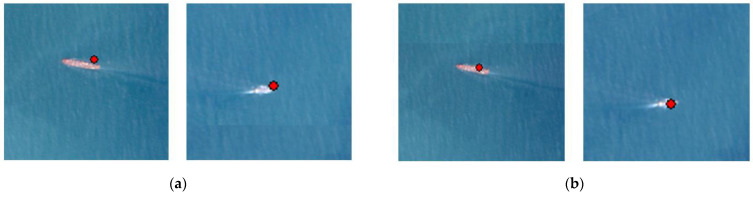
Display of correspondence between the remote-sensing image and the AIS data before and after positioning error correction. (**a**) Before positioning error correction; (**b**) after positioning error correction.

The comparison of the correspondence effect between the remote-sensing image and the AIS data before and after the correction of the positioning error is shown in Figure 16, where the red dots indicate the target positions provided by the AIS, whose positions are fixed. Through comparison, it can be observed that the ship targets in the image are closer to their real positions after correction, aligning with the calculated results and expectations from the checkpoints. This indicates that the approach of using AIS-provided positions as control points for correcting positioning errors in ship targets in remote-sensing images is feasible. In the case of fusion failure where only image data exists, the target can be able to be successfully targeted through its appearance and precise positioning information.

## 4. Conclusions

Addressing the practical demand for optical satellite ship-target monitoring and the challenge of precise geometric correction of remote-sensing images in remote oceanic areas where effective control points are difficult to obtain, this paper proposes a method to improve the accuracy of maritime abnormal target identification and localization based on the fusion of the AIS and remote-sensing images. To enhance the accuracy of target fusion, multiple-dimensional feature information of maritime targets is comprehensively considered. The optimal fusion between targets is achieved successively using the CPD algorithm and fuzzy comprehensive decision-making method, and the positional errors of targets are ultimately corrected using a polynomial model. The proposed algorithm exhibits low complexity and its reliability is demonstrated through experiments. Experimental results show that, compared to the original data, the positional accuracy of the final image is improved by over 70%. The positional deviations of 10 checkpoints in the *x* and *y* directions are significantly reduced, and the localization errors are effectively controlled within 4 pixels. In cases where fusion fails with only image data available, the image data provides more accurate positional information for such abnormal targets. This method offers a practical solution for maritime security by effectively enhancing the accuracy of identification and localization through the comprehensive utilization of multi-dimensional feature information, thereby providing crucial support for the safe and orderly conduct of maritime activities. It also shows promising prospects in the field of geometric correction of remote-sensing images in remote oceans. Moreover, the correlation fusion algorithm involved in the process can be applied to other types of data, providing a reference for research on data fusion technologies involving more sensors and higher levels.

Future research will focus on refining and improving the details of the overall process. Ship trails are important features of moving vessels, containing rich information such as physical, geometric, and optical characteristics, which provide important clues to the vessel’s motion state and behavior characteristics. Extracting ship trail information can not only assist in locating the geographical position of ship targets but also estimate key motion parameters such as heading and speed, which are of high research and practical value for achieving multi-faceted and large-scale remote-sensing monitoring of ship targets. Furthermore, the uniform distribution of control points affects the final accuracy of remote-sensing images. The complex marine environment, with scattered islands and large isolated islands, may pose constraints on control-point selection. Exploring the separation of sea and land and completing target-position correction in different regions is an interesting direction for further investigation.

## Figures and Tables

**Table 1:** Payload specifications of Jilin-1 Spectral-01 satellite.

Spectral Type	Spatial Resolution	Orbit Altitude	Orbit Inclination Angle	Revisit Period	Swath Width	Imaging Mode
Visible Light, Near Infrared	5 m	528 km	97.54°	2–3 days (for a twin satellite system)	Greater than 110 km	Push-broom, Night-light, Space target imaging
Short-wave Infrared, Mid-wave Infrared	100 m					
Long-wave Infrared	150 m					

**Table 2:** AIS static, dynamic, and voyage information.

Dynamic Information	Static Information	Voyage Information
Longitude and latitude	IMO number	Route planning
UTC	MMSI code	Port of destination
COG	Call sign and ship name	Ship draught
SOG	The length and width of the ship	Type of goods
Sailing condition	Antenna position	
ROT	Ship type	

**Table 3:** Ship-target fusion results based on fuzzy comprehensive decision-making.

Number of AIS Data	Number of Image Data	Number of Results	Evaluation Set Results
		142	Fusion success
265	647	123	Fusion failed, only the AIS data exists
		505	Fusion failed, only image data exists

**Table 4:** Partial summary information of successfully fused ship targets.

NO	MMSI	Ship Name	Ship Flag	Ship Type	Length/Width	Image
1	351460000	DD VICTORY	Panama	Cargo ship	159 m/27 m	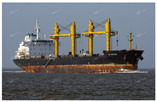
2	229006000	MALIK ALASHTAR	Malta	Container ship	366 m/48 m	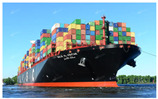
3	636018946	MIKE BAY	Liberia	Cargo ship	180 m/30 m	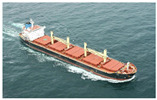
4	353111000	MSC SHANELLE V	Panama	Container ship	294 m/32 m	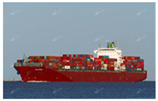

**Table 5:** Error values for 10 checkpoints.

No.	RMSE_x (m)	RMSE_y (m)	RMSE (m)
1	−1.69257	−2.96624	3.41517
2	−0.04506	−2.0298	2.0303
3	2.211928	−14.3563	14.52568
4	0.415481	−0.93009	1.018672
5	−0.01672	−1.55454	1.55463
6	−2.22911	−4.54245	5.059919
7	3.399991	−1.17741	3.598087
8	−1.28536	−8.51976	8.616174
9	−0.61811	−2.27077	2.353392
10	−0.56849	−2.34278	2.410767

## Data Availability

Data are contained within the article.

## References

[1] McCauley D.J., Woods P., Sullivan B., Bergman B., Jablonicky C., Roan A., Hirshfield M., Boerder K., Worm B. (2016). Ending Hide and Seek at Sea. Science.

[2] Fiorini M., Capata A., Bloisi D.D. (2016). AIS Data Visualization for Maritime Spatial Planning (MSP). Int. J. e-Navig. Marit. Econ..

[3] Fournier M., Casey Hilliard R., Rezaee S., Pelot R. (2018). Past, Present, and Future of the Satellite-Based Automatic Identification System: Areas of Applications (2004–2016). WMU J. Marit. Aff..

[4] Liang M., Liu R.W., Zhan Y., Li H., Zhu F., Wang F.-Y. (2022). Fine-Grained Vessel Traffic Flow Prediction With a Spatio-Temporal Multigraph Convolutional Network. IEEE Trans. Intell. Transport. Syst..

[5] Liu R.W., Liang M., Nie J., Lim W.Y.B., Zhang Y., Guizani M. (2022). Deep Learning-Powered Vessel Trajectory Prediction for Improving Smart Traffic Services in Maritime Internet of Things. IEEE Trans. Netw. Sci. Eng..

[6] Yan Q., Zheng S., Guan H., Li L. (2023). Association and Fusion of Ship AIS and Radar Track Data. J. Wuhan Univ. Technol..

[7] Xing X., Xie S., Huang M., Yang G., Huang S. (2020). A Target Fusion Algorithm Based on offshore Radar and AIS sensors Wave-Measuring Sensor Buoy. J. Ocean Technol..

[8] He F., Miu L., Tao F., Zhang C. (2017). A Method of Multi-Target Fusion and Tracking for Sea Surveillance Radar Based on AIS. Radar Sci. Technol..

[9] Zhao Z., Ji K., Xing X., Zhao H., Zhou S. (2013). Review of Ocean Surveillance Based on Information Fusion of Space-borne SAR and AIS. Mod. Radar.

[10] Song J., Kim D. (2021). Identification of Unclassified Ships Implementing AIS Information and SAR Image-Based Ship Detection Results. Proceedings of the 2021 IEEE International Geoscience and Remote Sensing Symposium IGARSS.

[11] Li H., Xiong W., Cui Y. (2023). An association method between SAR images and AIS information based on depth feature fusion. J. Syst. Eng. Electron..

[12] Liu Y., Yao L., Xiong W., Zhou Z. (2019). GF-4 Satellite and Automatic Identification System Data Fusion for Ship Tracking. IEEE Geosci. Remote Sens. Lett..

[13] Xuan G., Zhou C., Li Z., Xie H. (2023). Precise Geometric Correction Method for Oceanic Image Based on AIS Data for HEO Staring Satellites. Spacecr. Eng..

[14] Harati-Mokhtari A., Wall A., Brooks P., Wang J. (2007). Automatic Identification System (AIS): Data reliability and human error implications. J. Navig..

[15] Ren W., Zhou Z., Lyu S., Shi D. (2016). Position calibration technique of ship based on information of AIS. Syst. Eng. Electron..

[16] Pi Y., Xie B., Yang B., Zhang Y., Li X., Wang M. (2019). On-Orbit Geometric Calibration of Linear Push-Broom Optical Satellite Based on Sparse GCPs. Acta Geod. Cartogr. Sin..

[17] Pehani P., Čotar K., Marsetič A., Zaletelj J., Oštir K. (2016). Automatic Geometric Processing for Very High Resolution Optical Satellite Data Based on Vector Roads and Orthophotos. Remote Sens..

[18] Zhang Z., Shao Y., Tian W., Wei Q., Zhang Y., Zhang Q. (2017). Application Potential of GF-4 Images for Dynamic Ship Monitoring. IEEE Geosci. Remote Sens. Lett..

[19] Shorten C., Khoshgoftaar T.M. (2019). A Survey on Image Data Augmentation for Deep Learning. J. Big Data.

[20] Xu M., Yoon S., Fuentes A., Park D.S. (2023). A Comprehensive Survey of Image Augmentation Techniques for Deep Learning. Pattern Recognit..

[21] Lyu C., Huang H., Zhou Y., Wang Y., Liu Y., Zhang S., Chen K. (2022). An Empirical Study of Designing Real-Time Object Detectors. arXiv.

[22] Katsilieris F., Braca P., Coraluppi S., Boulevard M. (2013). Detection of Malicious AIS Position Spoofing by Exploiting Radar Information. Proceedings of the 16th International Conference on Information Fusion.

[23] Liu Y., Yao L., Wu Y., Xiu J., Zhou Z. (2018). Target Point Tracks Association and Error Correction with Optical Satellite in Geostationary Orbit and Automatic Identification System. J. Electron. Inf. Technol..

[24] Myronenko A., Song X. (2010). Point Set Registration: Coherent Point Drift. IEEE Trans. Pattern Anal. Mach. Intell..

[25] Min Z., Meng M.Q.-H. (2021). Robust and Accurate Nonrigid Point Set Registration Algorithm to Accommodate Anisotropic Positional Localization Error Based on Coherent Point Drift. IEEE Trans. Autom. Sci. Eng..

[26] Wang G., Chen Y. (2017). Fuzzy Correspondences Guided Gaussian Mixture Model for Point Set Registration. Knowl.-Based Syst..

[27] Xu X., Yu F., Pedrycz W., Du X. (2023). Multi-Source Fuzzy Comprehensive Evaluation. Appl. Soft Comput..

[28] Lu Y., Yamaoka F. (1997). Fuzzy Integration of Classification Results. Pattern Recognit..

[29] Zhu S., Zhou W., Zhang Y., Zhu B. (2004). Precision Comparison of Several Algorithms for Approximate Rectification of Linear Array Push-broom Imagery. J. Remote Sens..

[30] Pultarová I. (2007). Local Convergence Analysis of Iterative Aggregation–Disaggregation Methods with Polynomial Correction. Linear Algebra Its Appl..

